# Calcinosis and lipodystrophy: clinical presentation of 10 years non-treated juvenile dermatomyositis – case report

**DOI:** 10.1186/1546-0096-9-S1-P60

**Published:** 2011-09-14

**Authors:** Selmanovic-Mulaosmanovic Velma, Dizdarevic-Omercahic Aida, Mornjakovic-Franca Amela, Cengic Adisa

**Affiliations:** 1 Children’s Hospital, University Clinical Center Sarajevo, Bosnia and Herzegovina; 2 Institute of Radiology, University Clinical Center Sarajevo, Bosnia and Herzegovina

## Background

Association of dystrophic calcifications and lipodystrophy are mostly described in patients with juvenile dermatomyositis (JDM), only occasionaly as part of some other clinical entities. To our knowledge, these conditions are rarely reported as main reasons for first refferal to pediatric rheumatologist.

## Aim

Our goal is to demonstrate the benefit of basic and multidisciplinary diagnostic work up (specially imaging techniques) required for definitive diagnosis as in this case as well as therapy decision.

## Methods

We describe a 13,5 y old girl presented (april 2011) with extensive calcinosis and lipodystrophy, which were lasting for 10 years, without any treatment. General, musculoskeletal, pulmological and orthodontist evaluations were undertaken. Investigations included multiple radiographs of the calcified regions and chest X-ray, computed-tomography of the lungs, MRI of pelvis, coxofemoral and sacroiliacal joints.

## Results

Patient did not have typical clinical and laboratory signs required for definite JDM diagnosis, as is usually seen at disease onset, but history data confirmed that they existed 10 years ago. At the time of first referral to pediatric rheumatologist, she had extensive calcinosis (mixed forms), radiological signs of interstitial lung disease, mild pulmonary hypertension, osteopenia, multiple osteofits and lateroghenia.

## Conclusion

Distinction between muscle and skin inflammation and residual state can be difficult expecially in case of probably monocyclic course of muscle disease. Multidisciplinary diagnostic work up is ultimate in process of therapy decision in case of 10 years non-treated juvenile dermatomyositis.

